# Sustained and durable response with Alisertib monotherapy in the treatment of relapsed Atypical Teratoid Rhabdoid Tumor (ATRT)

**DOI:** 10.1093/noajnl/vdac090

**Published:** 2022-06-07

**Authors:** Kaitlyn Howden, Patrick J McDonald, Colin Kazina, Annie Ong, Ben Ho, Annie Huang, Brent A Orr, Magimairajan Issai Vanan

**Affiliations:** Department of Pediatrics and Child Health, University of Manitoba, Manitoba, Canada; Department of Pediatrics and Child Health, University of Manitoba, Manitoba, Canada; Section of Neurosurgery, University of Manitoba, Winnipeg, Manitoba, Canada; Department of Pediatrics and Child Health, University of Manitoba, Manitoba, Canada; Section of Neurosurgery, University of Manitoba, Winnipeg, Manitoba, Canada; Department of Pharmacy, Cancer Care Manitoba, Manitoba, Canada; Department of Pediatrics, University of Toronto, Toronto, Ontario, Canada; Division of Hematology-Oncology, Hospital for Sick Children, Toronto, Ontario, Canada; Department of Pediatrics, University of Toronto, Toronto, Ontario, Canada; Division of Hematology-Oncology, Hospital for Sick Children, Toronto, Ontario, Canada; Department of Pathology, St Jude Children’s Research Hospital, Memphis, Tennessee, USA; Department of Pediatric Hematology & Oncology, Cancer Care Manitoba, Manitoba, Canada; Department of Pediatrics and Child Health, University of Manitoba, Manitoba, Canada; Cancer Care Manitoba Research Institute, University of Manitoba, Manitoba, Canada

**Keywords:** Alisertib, ATRT, Aurora kinase A, monotherapy, relapsed

Atypical teratoid rhabdoid tumors (ATRT) account for 1–2% of all primary central nervous system (CNS) malignancies in children^1^ and are the most common CNS tumors in infants.^1,2^ Various combinations of therapeutic approaches including surgery, followed by high dose chemotherapy regimens and craniospinal radiation have not had a major effect on outcomes with a 2-year OS of less than 50% in this population.^1–4^ ATRTs are distinguished by the presence of biallelic inactivation of SWItch/sucrose nonfermentable (SWI/SNF) related, matrix associated, actin dependent regulator of chromatin, subfamily B1 (SMARCB1).^2^ One of the downstream targets of SMARCB1 is Aurora kinase A (AURKA), an oncoprotein that plays an important role in the bipolar spindle apparatus during mitosis and regulation of the spindle checkpoint during the cell cycle.^5^ Alterations of SMARCB1 have been associated with loss of AURKA regulation and over-expression, which has known oncogenic effects due to the development of genomic instability.^4,6^ Alisertib (MLN8237) is a specific AURKA inhibitor that has been tried in the treatment of various adult cancers like non-Hodgkin’s lymphoma, breast and lung cancers.^5,7,8^

## Case Report

**Figure 1.**
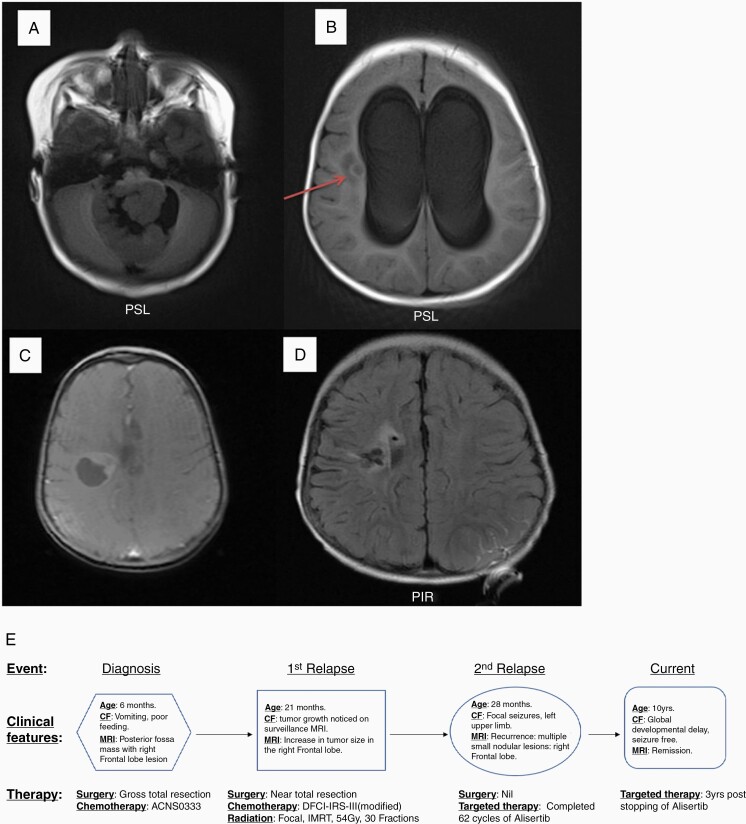
Axial T1 MRI images of the brain (A) showing posterior fossa mass measuring 4.4 cm (AP) × 2.1 cm (CC) × 4.0 cm (T) and nodule (B) measuring 0.8 cm in the right periventricular white matter with evidence of obstructive hydrocephalus. Axial T1 postcontrast MRI images at first relapse in the right frontal lobe (C) and at second relapse with increase in the size of the tumor in the right frontal lobe (D). (E) shows the graphical summary of the management of our patient from diagnosis through the two relapses.

**Figure 2.**
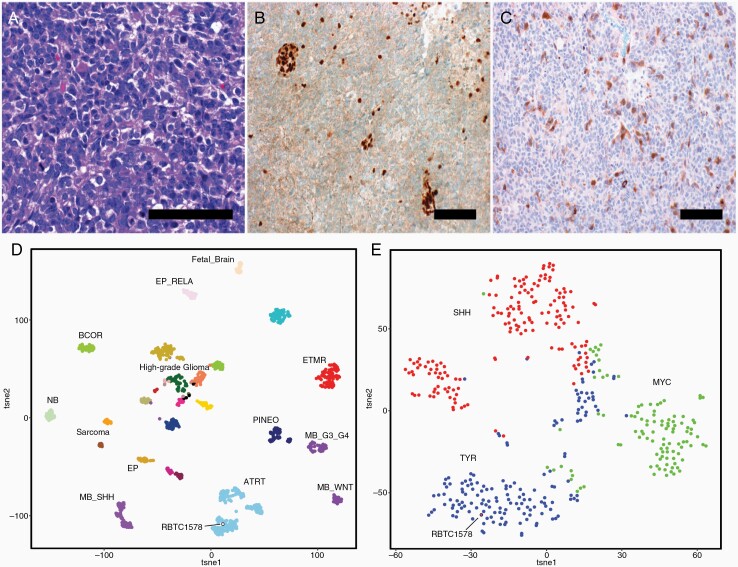
Immuno-histochemistry: H&E stain showing tumor cells with vacuolated cytoplasm and rhabdoid cells (A). There was loss of INI-1 staining in tumor cells with retention in nonneoplastic endothelial cells (B). The tumor demonstrated immunoreactivity with antibodies to Aurora kinase A in a cytoplasmic and nuclear distribution (C). Unsupervised clustering of reference cohort samples (*n *= 1200) against target sample (RBTC1578, clustering in the ATRT cohort) by t-distributed stochastic neighbor embedding (tSNE) dimensionality reduction analysis. Individual samples are labeled in the respective tumor class (D). Close up visualization of target sample in ATRT cluster (*n *= 427) – showing the patient sample (RBTC1578) clustering in the TYR sub-group (E). Scale bar in A, B, and C = 100 microns.

A six-month-old female patient presented to the emergency department with a six-week history of vomiting, poor feeding, and progressive lethargy. Clinical exam revealed macrocephaly with sun setting sign and bulging fontanelle. Magnetic resonance imaging (MRI) of the brain showed a posterior fossa mass measuring 4.4 cm × 2.1 cm × 4.0 cm with associated obstructive hydrocephalus (Figure 1A). A small 0.8 cm nodule within the periventricular white matter near the right lateral ventricle suspicious for metastasis was also noted (Figure 1B). She underwent gross total resection (GTR) of the posterior fossa mass and required VP shunting for obstructive hydrocephalus after tumor resection. Pathology revealed findings consistent with ATRT: tumor cells with vacuolated cytoplasm and rhabdoid cells (Figure 2A) and loss of INI-1 staining in tumor cells with retention in nonneoplastic endothelial cells (Figure 2B). MRI of the spine and CSF analysis was negative for metastasis. Ultrasound of the abdomen revealed normal kidneys. There was no family history of cancer. She was initially treated as per COG-ACNS0333 protocol and tolerated the chemotherapy without any complications.^9^ Fifteen months after completion of chemotherapy she relapsed in the right frontal lobe (Figure 1C). She underwent sub-total resection of the tumor and pathology confirmed relapse of ATRT: densely cellular, poorly differentiated neoplasm with solid growth pattern, areas of necrosis, and a high mitotic index (30–40%) with no evidence of any therapy related changes seen in this tumor. The tumor cells showed highly pleomorphic nuclei and many rhabdoid cells with eccentric nuclei with loss of INI1 expression. Therapy included Focal Radiation (IMRT) – 54 Gy/30 fractions, Chemotherapy (DFCI-IRS-III, modified protocol) Doxorubicin/Etoposide alternating with Actinomycin-D, and triple intra-thecal chemotherapy (Methotrexate 15 mg/m^2^, Hydrocortisone 30 mg/m^2^, Cytarabine 60 mg/m^2^). After completing 7 months of therapy, she presented with focal seizures of the left upper limb and MRI revealed an increase in the size of the tumor in the right frontal lobe (Figure 1D). Our patient’s original tumor at diagnosis was positive for AURKA by immuno-histochemistry (Figure 2C), and based on evidence in the literature (both preclinical/clinical)^5,7,8,10^ of efficacy of Alisertib (MLN8237) in the treatment of relapsed ATRT, she was treated with a trial of Alisertib monotherapy (60 mg/m^2^ by mouth once daily for 7 days of a 21-day treatment cycle). She was in remission after 8 months (11 cycles) of treatment. The main side effects experienced by the patient while on Alisertib were increased daytime somnolence as well as intermittent episodes of neutropenia, which were managed with administration of the drug at bedtime and observation without any dose modifications respectively. Given the lack of data suggesting optimal treatment duration, Alisertib was continued for 3 years and gradually tapered (one week on, 3 weeks off for 9 months, followed by one week on, 4 weeks off for 4 months) and stopped after a total of 4 years of therapy (a total of 62 cycles). She has been in remission for over 3 years since stopping Alisertib. Along with radiological remission, our patient experienced significant clinical improvement while on Alisertib. Initially, she had significant right sided motor weakness and hearing loss associated language delays secondary to her tumor and complications from her prior treatment course. While she still has severe global developmental delay, she has made significant gains throughout her treatment course while on Alisertib. She is able to walk independently and has improved her language skills with the help of hearing aids and is able to attend school on a regular basis. Summary of her treatment is shown in Figure 1E.

### Pathology and Molecular Diagnostics

Our patient was screened for rhabdoid tumor predisposition syndrome (RTPS), and cytogenetic analysis (Dr J Biegel, Children’s Hospital of Los Angeles) using multiplex-ligation dependent probe amplification (MLPA) in the tumor sample demonstrated a heterozygous deletion of chromosome arm 22q and a homozygous deletion of exons 8–9 of the SMARCB1 gene. The blood sample demonstrated a heterozygous deletion of exons 8–9 of SMARCB1. Parental testing did not reveal exons 8–9 deletion in both parents. This could be likely explained by germline gonadal mosaicism. Immuno-histochemistry for INI1 (BT Transduction Laboratories, clone 25/BAF47, 1:200) and AURKA (Abcam, clone EP1008Y, 1:100) was performed on deparaffinized sections from the tumor sample at diagnosis in a CLIA approved lab (St Jude Children’s Research Hospital, B.A.O.). AURKA immunoreactivity was scored as positive due to the presence of nuclear and cytoplasmic immunoreactivity in the tumor cells (Figure 2C). Due to the small size of the tumor and very limited tumor tissue available at 1st relapse we did not test the relapsed tumor for AURKA expression or methylation studies. Molecular subgrouping of the primary tumor tissue at diagnosis using DNA methylation arrays showed that our patient had ATRT-TYR subgroup (Figure 2D and E). Briefly, sample DNA was processed for global methylation profiling analyses on the Illumina 850K platform (Illumina, San Diego, CA) using published methods at the University Health Network Genomics Core.^11^ Processing and analyses of methylation data were performed as previously reported.^9,11^ Briefly, the methylation profile was first analyzed relative to a reference set of 1200 pediatric brain tumors to confirm diagnosis as ATRT and then in relation to a methylation data set of 427 primary ATRTs to establish subtype. Consensus subgroup was obtained from unsupervised t-distributed stochastic neighbor embedding (tSNE) dimensionality reduction analysis and hierarchical clustering analysis. For the analysis against primary ATRTs, additional analysis was performed using the non-negative matrix factorization (NMF) algorithm. Finally, the sample subgroup was independently verified against the methylation brain classifier (MNP v11b2) with prediction score > 0.90.^12^

## Discussion

ATRTs have remained a challenging tumor subtype to manage despite many years of progress in our understanding of them and evolution in our treatment approaches. The current consensus classification identifies 3 molecular subgroups of ATRT (ATRT-TYR, ATRT-SHH, and ATRT-MYC) with distinct molecular, clinical and prognostic features.^11^ The ATRT-TYR subgroup is named after the enzyme tyrosinase, which is specifically overexpressed in this subgroup. Our patient had many characteristics of the TYR subgroup including age at presentation (<1 yr), tumor location (infratentorial), and genetic findings. Several subgroup specific drug targets have been described in the ATRT-TYR subgroup including platelet derived growth factor receptor B (PDGFRB), fibroblast growth factor receptor 2 (FGFR2), and Janus kinase 1 (JAK1)^11^ and there are ongoing clinical trials using some of these agents.^4^

Based on the findings from the EU-RHAB study,^13^ our patient falls into the intermediate risk category at diagnosis (<1 yr + ATRT-TYR), although our patient had several high-risk factors like metastatic tumor, germline mutation, and omission of radiation at initial treatment. To date, there have been few studies looking into the use of Alisertib in AURKA positive solid tumors, and even fewer looking into its use specifically for pediatric patients with ATRT. One study out of St Jude Children’s Research Hospital looking at pediatric patients diagnosed over a decade ago with ATRT and treated with Alisertib showed promising results, with all 4 patients having stabilization or decreased size of tumor after only 1–2 cycles of treatment, and a median duration of 11 months for disease stability (Table 1).^10^ While two of the patients progressed while on Alisertib, leading to discontinuation of the medication, two were still on treatment years after starting.^10^ In this study, the Alisertib dosing was started on 80 mg/m^2^/day and dropped to 60 mg/m^2^/day only if the higher dose was not tolerated, whereas our patient was started on the lower dose from the beginning; otherwise, the dosing cycles used for our patient was similar.^10^ The most common side effects experienced by both this cohort and our patient, which is congruent with effects described in the adult literature, were increased daytime somnolence due to the benzodiazepine-like effect of this medication and bone marrow suppression with neutropenia.^7,8,10^ Outside of this small cohort, to our knowledge there have not been other cases published demonstrating the effectiveness of Alisertib in pediatric ATRT patients. The actual treatment duration with Alisertib, the time that a patient can remain off and maintain disease stability, and the long-term effects of being on therapy are still not clear from the limited literature that is available and requires future exploration with larger patient populations. After 3 years of regular dosing treatment, we started tapering the drug and stopped after 1 year. Presently it is difficult to explain why certain patients do well on this form of targeted therapy while others fail after a period of time, such as the two patients in the St Jude cohort.^10^ Germline mutations and non-TYR subgroup are known to be poor prognostic factors in ATRT.^13^ Since we were not able to determine the germline status or ATRT subgroup of patients in the St Jude series, it is difficult to comment on correlation between germline inheritance pattern or ATRT subgroup and response to Alisertib. Immunohistochemistry staining of our patient’s tumor cells showed only a moderate proportion expressed AURKA (Figure 2C), with adult studies supporting the idea that AURKA expression does not correlate with clinical benefit with use of Alisertib.^7^ This is likely explained by factors related to response to Alisertib induced cell death (length of mitotic arrest and activation of apoptotic pathways) being independent of AURKA expression and possible inhibition of AURKB (Aurora kinase B) in addition to AURKA by Alisertib.^5^ Finally, what also is unclear at this time is whether targeted therapies like Alisertib would be appropriate to consider as upfront treatment versus current chemotherapeutic and radiation regimens. There is no evidence for its up-front use in both adults and pediatrics, as past studies have only used it in relapsed populations. However, there is an ongoing Phase-II clinical trial (NCT02114229) using single agent Alisertib in the relapsed/progressive setting and as combination with age and risk adapted chemotherapy in newly diagnosed patients. Data from these trials will assist in determining if this approach can improve clinical outcomes for this population of cancer patients.

**Table 1.** Alisertib Monotherapy in Relapsed ATRT – Literature Review

Patient #/Age at Diagnosis/Sex	Tumor Location/Extent of Resection	Radiation (Type/Dose-Gy)	Site of Recurrence	Alisertib Cycles	Best Response	Comments
1/1.39/M	RF/STR	F/55.8	Right thalamus	5	SD	Alive
2/4.87/F	PF/GTR	CSI/(23.4 + 55.8)	Spine	23	PR	Dead
3/3.26/F	PF/GTR	CSI/(23.4 + 55.8)	Spine/CSF	14	PR	Alive
4/1.75/M	Pineal/GTR	F (Protons)/59.4	Brainstem	34	CR	Dead
5/0.6/F	PF/RF/GTR	F/54	RF	62	CR	Alive

Abbreviations: CR, complete response; CSI, cranio-spinal irradiation; F, female; F, focal; GTR, gross total resection, Gy, gray; M, male; PF, posterior fossa; PR, partial response; RF, right frontal lobe; SD, stable disease; STR, sub-total resection.

## Conclusion

In summary, ATRT comprises a difficult subset of CNS tumors to manage in pediatric patients, and there exists a lack of progress in patient outcomes that requires novel treatment approaches. While limited conclusions can be drawn from the small sample size of patients that exists in the pediatric literature, our case report adds to the literature of how targeted therapies against AURKA can be effective to cause radiologic stability and clinical improvement in patients with ATRT with tolerable side effects. Single agent Alisertib in the treatment of relapsed ATRT in the TYR subgroup produces marked and durable regression in tumor burden. Alisertib is tolerated well with minimal side effects and is safe to administer long term in children. Ongoing clinical trials involving Alisertib monotherapy in ATRT in the relapsed setting or in combination with conventional chemotherapy at diagnosis will help validate our findings.

## Notes

**Authorship statement.** P.J.M., C.K., A.O. and M.I.V. diagnosed and managed the patient. K.H. and M.I.V. wrote the manuscript and prepared the data, text and figures. B.H. and A.H. helped in molecular sub-grouping of the tumor and B.A.O. helped in AURKA analysis of the tumor. All authors read, edited and approved the final manuscript.

## References

[1] Ginn K , Gajjar A. Atypical teratoid rhabdoid tumor: current therapy and future directions. Front Oncol. 2012; 2(114):1–13. 10.3389/fonc.2012.00114 PMC3439631 22988546

[2] Fruhwald M , Biegel J, Bourdeaut F, Roberts C, Chi S. Atypical teratoid/rhabdoid tumors - current concepts, advances in biology, and potential future therapies. Neuro Oncol. 2016; 18(6):764–778. 26755072 10.1093/neuonc/nov264 PMC4864253

[3] Lafay-Cousin L , Hawkins C, Carret AS, et al Central nervous system atypical teratoid rhabdoid tumors: the Canadian Pediatric Brain Tumor Consortium experience. Eur J Cancer. 2012; 48(3):353–359. 22023887 10.1016/j.ejca.2011.09.005

[4] Hoffman L , Richardson E, Ho B, et al Advancing biology-based therapeutic approaches for atypical teratoid rhabdoid tumors. Neuro Oncol. 2020; 22(7):944–954. 32129445 10.1093/neuonc/noaa046 PMC7339881

[5] Hilton J , Shapiro G. Aurora kinase inhibition as an anticancer strategy. J Clin Oncol. 2014; 32(1):57–59. 24043748 10.1200/JCO.2013.50.7988

[6] Nesvick C , Lafay-Cousin L, Raghunathan A, et al Atypical teratoid rhabdoid tumor: molecular insights and translation to novel therapies. J Neurooncol. 2020; 150(1):47–56. 33021733 10.1007/s11060-020-03639-w PMC8230985

[7] Friedberg J , Mahadevan D, Cebula E, et al Phase II study of alisertib, a selective aurora A kinase inhibitor, in relapsed and refractory aggressive B- and T-cell non-Hodgkin lymphomas. J Clin Oncol. 2013; 32(1):44–50. 24043741 10.1200/JCO.2012.46.8793 PMC3867644

[8] Melichar B , Adenis A, Lockhart C, et al Safety and activity of alisertib, an investigational aurora kinase A inhibitor, in patients with breast cancer, small-cell lung cancer, non-small-cell lung cancer, head and neck squamous-cell carcinoma, and gastro-oesophageal adenocarcinoma: a five-arm phase 2 study. Lancet Oncol. 2015; 16(4):395–405. 25728526 10.1016/S1470-2045(15)70051-3

[9] Reddy A , Strother D, Judkins A, et al Efficacy of high-dose chemotherapy and three-dimensional conformal radiation for atypical teratoid/rhabdoid tumor: a report from the Children’s Oncology Group Trial ACNS0333. J Clin Oncol. 2020; 38(11):1175–1185. 32105509 10.1200/JCO.19.01776 PMC7145589

[10] Wetmore C , Boyett J, Li S, et al Alisertib is active as single agent in recurrent atypical teratoid rhabdoid tumors in 4 children. Neuro Oncol. 2015; 17(6):882–888. 25688119 10.1093/neuonc/nov017 PMC4483126

[11] Ho B , Johann P, Grabovska Y, et al Molecular subgrouping of atypical teratoid/rhabdoid tumors - a reinvestigation and current consensus. Neuro Oncol. 2020; 22(5):613–624. 31889194 10.1093/neuonc/noz235 PMC7229260

[12] Capper D , Jones D, Sill M, et al. DNA methylation-based classification of central nervous system tumors. Nature. 2018; 555(7697): 469–474. 29539639 10.1038/nature26000 PMC6093218

[13] Fruhwald MC , Hasselblatt M, Nemes K, et al. Age and DNA methylation subgroup as potential independent risk factors for treatment stratification in children with atypical teratoid/rhabdoid tumors. Neuro Oncol. 2020; 22(7): 1006–1017. 31883020 10.1093/neuonc/noz244 PMC7339901

